# Physical activity and reduced risk of fracture in thyroid cancer patients after thyroidectomy — a nationwide cohort study

**DOI:** 10.3389/fendo.2023.1173781

**Published:** 2023-07-20

**Authors:** Jinyoung Kim, Kyungdo Han, Jin-Hyung Jung, Jeonghoon Ha, Chaiho Jeong, Jun-Young Heu, Se-Won Lee, Jeongmin Lee, Yejee Lim, Mee Kyoung Kim, Hyuk-Sang Kwon, Ki-Ho Song, Ki-Hyun Baek

**Affiliations:** ^1^ Division of Endocrinology and Metabolism, Department of Internal Medicine, Yeouido St. Mary’s Hospital, College of Medicine, The Catholic University of Korea, Seoul, ;Republic of Korea; ^2^ Department of Statistics and Actuarial Science, Soongsil University, Seoul, ;Republic of Korea; ^3^ Division of Endocrinology and Metabolism, Department of Internal Medicine, Seoul St. Mary’s Hospital, College of Medicine, The Catholic University of Korea, Seoul, ;Republic of Korea; ^4^ Division of Endocrinology and Metabolism, Department of Internal Medicine, Uijeongbu St. Mary’s Hospital, College of Medicine, The Catholic University of Korea, Seoul, ;Republic of Korea; ^5^ Department of Orthopedic Surgery, Yeouido St. Mary’s Hospital, College of Medicine, The Catholic University of Korea, Seoul, ;Republic of Korea; ^6^ Division of Endocrinology and Metabolism, Department of Internal Medicine, Eunpyeong St. Mary’s Hospital, College of Medicine, The Catholic University of Korea, Seoul, ;Republic of Korea; ^7^ Division of General Internal Medicine, Department of Internal Medicine, Seoul National University Bundang Hospital, Seoul National University College of Medicine, Seoul, Republic of Korea

**Keywords:** thyroid cancer, cancer survivorship, fracture, physical activity, prevention and control

## Abstract

**Objectives:**

Levothyroxine suppressive therapy following thyroidectomy for thyroid cancer patients is considered as a risk factor for osteoporosis and fragility fractures. We evaluated the association of regular exercise and exercise habit change with fracture risk in adults older than 40 years who underwent thyroidectomy for thyroid cancer.

**Methods:**

We enrolled the patients who underwent thyroidectomy for thyroid cancer older than 40 years between 2010 and 2016 from the Korean National Health Insurance Service data, and they were followed through 2019. Based on the questionnaire of health examination within 2 years before and after surgery, whether regular exercise once a week was evaluated. The reference group for the statistical analysis was the continuing lack of physical activity group that did not exercise before or after surgery. For fractures newly diagnosed during the follow-up period, univariate and multivariate Cox regression analyses were performed for risk evaluation.

**Results:**

We evaluated 74,774 subjects, of whom 2,924 (3.9%) experienced any fractures during a median follow-up of 4.5 years. Compared with the group consistently lack of physical activity, the group that exercised before and after surgery showed a significant decrease in the risk of any fracture, vertebral fracture, and hip fracture: adjusted hazard ratio 0.848 (95% Confidence Interval 0.771–0.932), 0.703 (0.591–0.836), and 0.405 (0.224–0.732), respectively. For vertebral fracture, a significant reduction in fracture risk was confirmed even in patients who started their regular exercise after surgery: adjusted hazard ratio 0.779 (0.648–0.936). The risk reduction for vertebral fractures upon the initiation of exercise was found to be significant in the high-risk groups of patients: women and total thyroidectomy patients.

**Conclusion:**

We suggest that maintaining or starting regular exercise after surgery may help prevent fractures in thyroid cancer patients older than 40 years who have undergone thyroidectomy.

## Introduction

Most patients with thyroid cancer have a good prognosis with a 5-year survival rate as high as 97% (1), so it is important to minimize the treatment-related harmful effects in treating thyroid cancer (2), Levothyroxine replacement after thyroidectomy is essential for patients who underwent total thyroidectomy, and it has been reported that about 47-62% of patients require levothyroxine replacement even after lobectomy (3, 4). In association with long-term levothyroxine replacement, bone health is an important consideration along with cardiovascular diseases such as atrial fibrillation (5, 6).

Thyroid hormone excess is known to cause bone loss by accelerating bone turnover (7). In addition to the effects of thyroid hormone itself, previous studies suggested that thyroid stimulating hormone (TSH) acts on bone metabolism independently of thyroid hormone. TSH receptor expression has been identified in both osteoblast and osteoclast, and it acts especially on the differentiation and function of osteoblast (8). Therefore, TSH suppression can also lead to bone weakness by shifting the balance of bone remodeling towards resorption by weakening the action of osteoblast (9). In patients who received thyroidectomy as part of the treatment for differentiated thyroid cancer, levothyroxine at doses high enough to suppress TSH is recommended according to the guidelines of the American Thyroid Association (10). Therefore, thyroid cancer patients underwent surgery are considered to be a high-risk group for osteoporosis and fragility fractures, and special attention to their bone health is needed.

Meanwhile, increasing physical activity is a modifiable factor for reducing the risk of fractures, and studies have reported that regular exercise is effective in preventing fragility fractures (11). Physical activity can increase bone mass and has other beneficial effects on musculoskeletal health, such as maintaining muscle strength and exercise ability to cope with falls (12, 13). However, most of the previous studies reporting the beneficial effects of exercise on bone health were conducted on elderly patients, and there were few studies on thyroid cancer survivors receiving levothyroxine replacement.

Therefore, we aimed to investigate the association of regular exercise and exercise habit change on fracture risk in adults older than 40 years who underwent thyroidectomy for thyroid cancer.

## Materials and methods

### Study design

The Korean National Health Insurance covers the almost all citizens in the country, and about 20% of the population undergoes regular health examinations every two years (14). This study was designed based on this nationwide health check-up data, and diagnosis and surgery codes for insurance claims were additionally used. For subject screening, people with claims for thyroid surgery were extracted from the claims data using the operating codes: total thyroidectomy (P4561 and P4552) and lobectomy (P4551, P4553, and P4554). To confirm each patient’s surgical purpose, we determined whether the thyroid cancer code (C73) was applied for reimbursement of the costs of thyroidectomy within one year prior to surgery.

### Study population

**Figure 1:**
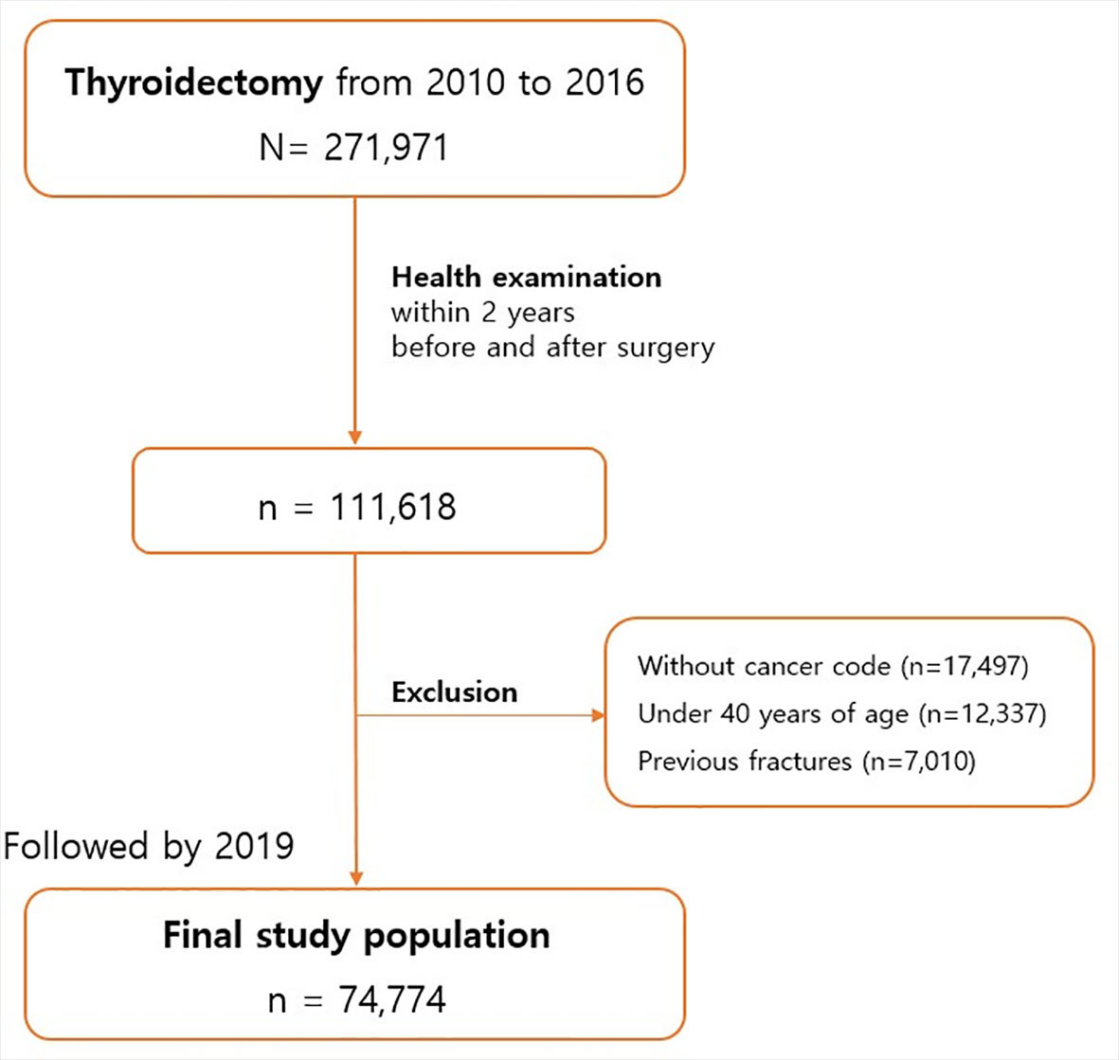
Study Flow.

This population-based observational study analyzed patients who requested imbursement for thyroidectomy from the Korean National Health Insurance Service between 2010 and 2016. Of the 271,971 patients who underwent thyroidectomy, we selected those who received regular health check-ups from the insurance service before and after surgery. Among the 111,618 patients with sufficient data, we excluded non–thyroid cancer patients (n=17,497), those younger than 40 years (n=12,337), and those with a previous fracture history (n=7,010) (**Figure 1**). The final study population thus totaled 74,774 patients. The last date of follow-up was December 31, 2019, and the median follow-up period was 4.5 years. The protocol of this study was reviewed by the institutional review board of Yeouido St. Mary’s Hospital (SC22ZISE0150).

### Measurements and definitions

Each health check-up includes physical measurements (height, weight, waist circumference), blood pressure measurement, blood tests, and self-reported questionnaires for evaluating life-style factors. Diabetes mellitus is defined as a fasting glucose level of 126 mg/dL or higher or the use of anti-diabetes medication; hypertension is a systolic blood pressure over 140 mmHg or diastolic blood pressure over 90 mmHg or the use of antihypertensive drugs; hyperlipidemia is a total cholesterol level of 240 mg/dL or higher or the used of anti-hyperlipidemia medication. Chronic kidney disease (CKD) is defined as an estimated glomerular filtration rate, measured using the modification of diet in renal disease equation, of less than 60. Regular exercise was defined as moderate to vigorous physical activity (jogging, running, biking, and sports such as tennis, basketball, or soccer) at least one day per week. The questionnaire to investigate the degree of physical activity was written in Korean and self-reported; an English version of these questions is presented in **Supplementary Table ST1**.

The endpoint for fracture risk was newly diagnosed fractures at typical skeletal sites of osteoporotic fractures: vertebrae and hip (15). Vertebral fracture was identified as two or more outpatient visits with ICD-10 codes of S22.0, S22.1, S32.0, M48.4, or M48.5 within 12 months. Hip fracture was defined as hospitalization with the relevant ICD-10 codes (S72.0 and S72.1). Any fractures were defined as vertebrae, hip, or other common fracture sites including the clavicle (S42.0), upper arm (S42.2, and S42.3), wrist (S52.5, and S52.6), or ankle (S82.3, S82.5 and S82.6). Traumatic fractures are usually caused by accidents and are covered separately by automobile or industrial insurance in Korea. Therefore, we assumed that traumatic fractures were excluded from the dataset we used.

### Statistical analysis

Descriptive statistics are expressed as mean values and standard deviations for continuous variables, and as numerical values and percentages for categorical variables. Comparisons between groups were made using t-tests and chi-square tests, for continuous and categorical variables, respectively. The study population was stratified into four groups based on physical activity levels before and after surgery, as shown by the results of health check-ups within two years before and after surgery. The incidence rate (new cases per 1000 person-years) of any fracture was calculated according to the physical activity group. Hazard ratios (95% confidence intervals) were estimated with multivariate Cox regression analysis. The reference group for the statistical analysis was the continuing lack of physical activity group that did not exercise before or after surgery. For fractures newly diagnosed during the follow-up period, univariate and multivariate Cox regression analyses were performed for risk evaluation. All statistical analyses were performed using SAS version 9.4. (SAS Institute Inc., Cary, NC, USA).

## Results

### Baseline characteristics of the study population

Among the 74,774 study subjects, 2,924 (3.9%) experienced any fractures, of which 1,017 (1.4% of the study population) were vertebral or hip fractures during a median follow-up duration of 4.5 years. The group who experienced fractures was older than the group that did not (mean age ± standard deviation was 59 ± 9 in fracture group and 53 ± 9 in non-fracture group, p<0.001), and the proportion of women was higher (90% in fracture group and 78% in non-fracture group, p < 0.001). The group with newly diagnosed fractures had a larger waist circumference and higher proportion of metabolic disorders (diabetes mellitus, hypertension, and hypercholesterolemia) and CKD than the group without fractures (**Table 1**). When we compared the incidence of fractures according to surgical methods, we found that fractures occurred more frequently in the total thyroidectomy group than the lobectomy group (4.2% in total thyroidectomy group and 2.9% in lobectomy group, p < 0.001).

**Table 1:** Baseline characteristics of the study population.

	Total	Any Fracture		*P*
		Yes	No	
Number	74,774	2,924 (3.9)	71,850 (96.1)	
Age, years (mean±sd)	53.55 ± 8.64	58.81 ± 8.51	53.34 ± 8.58	< 0.001
Sex, female (n, %)	58,788 (78.62)	2,640 (90.29)	56,148 (78.15)	< 0.001
Height, cm (mean±sd)	159.88 ± 7.86	157.28 ± 6.7	159.99 ± 7.89	<0.001
Weight, kg (mean±sd)	62.07 ± 10.72	60.25 ± 9.42	62.14 ± 10.76	<0.001
BMI, kg/m^2^	24.20 ± 3.26	24.32 ± 3.21	24.20 ± 3.26	0.056
WC, cm (mean±sd)	79.92 ± 8.96	80.46 ± 8.65	79.90 ± 8.97	0.001
Fasting glucose, mg/dL (mean±sd)	98.96 ± 20.05	100.96 ± 24.04	98.88 ± 19.86	<0.001
DM (n, %)	8,249 (11.03)	462 (15.8)	7,787 (10.84)	<0.001
Systolic BP, mmHg (mean±sd)	121.8 ± 14.08	123.85 ± 14.27	121.71 ± 14.06	<0.001
Diastolic BP, mmHg (mean±sd)	75.91 ± 9.51	76.38 ± 9.37	75.90 ± 9.52	0.007
HTN (n, %)	25,450 (34.04)	1,309 (44.8)	24,141 (33.6)	<0.001
Total cholesterol, mg/dL (mean±sd)	192.61 ± 36.72	191.23 ± 37.38	192.67 ± 36.69	0.039
Hyperlipidemia (n, %)	21,172 (28.31)	990 (33.86)	20,182 (28.09)	<0.001
GFR, mL/min/1.73m^2^ (mean±sd)	92.14 ± 34.81	90.63 ± 31.34	92.2 ± 34.94	0.017
CKD (n, %)	2,821 (3.77)	178 (6.09)	2,643 (3.68)	<0.001
Income, low (n, %)	13,618 (18.21)	579 (19.80)	13,039 (18.15)	0.023
Surgery - TTx (n, %) - Lobectomy (n, %)	58,193 (77.83) 16,581 (22.17)	2,441 (4.2) 483 (2.9)	55,752 (95.8) 16,098 (97.1)	<0.001

BMI, body-mass index; WC, waist circumference; DM, diabetes mellitus; BP, blood pressure; HTN, hypertension; GFR, glomerular filtration rate; CKD, chronic kidney disease; TTx, total thyroidectomy.

### Risk of fracture according to interval changes in regular exercise

**Figure 2:**
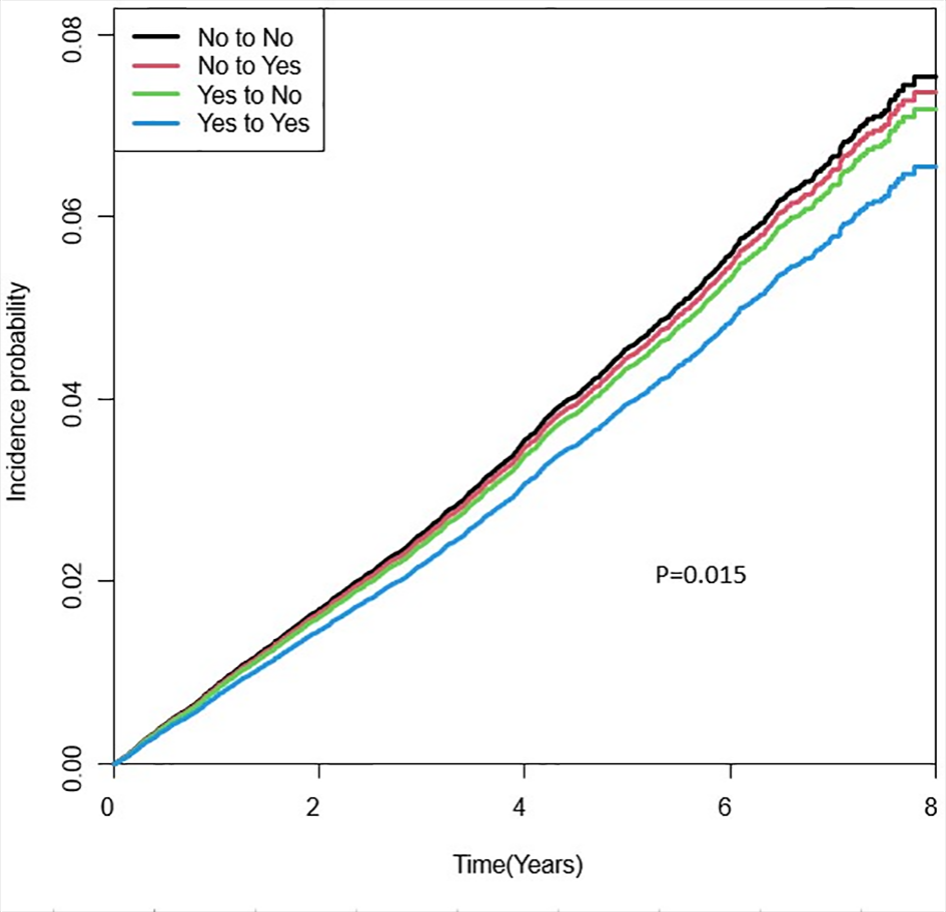
Cumulative Incidence Probability of Any Fracture According to the Physical Activity Group.

The incidence rate (new cases of per 1000 person-years) of any fracture was 10.9 in the continuous lack of physical activity group, 9.1 in the group started exercise after surgery, 9.4 in the group stopped exercise after surgery, and 6.8 in the group continued to exercise. Compared with patients who did not exercise, the group who exercised both before and after surgery had a significantly lower risk of any fracture, vertebral fracture, and hip fracture (**Figure 2**). After adjusting for all relevant variables, including surgery type, the adjusted hazard ratios for any fracture, vertebral and hip fractures were 0.848 (95% confidence interval 0.771–0.932), 0.703 (0.591–0.836), and 0.405 (0.224–0.732), respectively. For vertebral fracture, a significant reduction in fracture risk was confirmed even in patients who started their regular exercise after surgery: adjusted hazard ratio 0.779 (0.648–0.936) (**Table 2**).

**Table 2:** Univariate and multivariate cox-proportional hazard models for fractures.

Physical Activity Change	Number	Event	Duration (days)	Rate*	Model 1	Model 2	Model 3
Any Fracture							
No → No	19,844	960	88,261	10.88	1 (reference)	1 (reference)	1 (reference)
No → Yes	15,652	642	70,658	9.09	0.834 (0.755,0.922)	0.965 (0.873,1.067)	0.967 (0.875,1.069)
Yes → No	11,849	500	52,948	9.44	0.868 (0.779,0.967)	0.942 (0.845,1.049)	0.942 (0.845,1.049)
Yes → Yes	27,429	822	121,698	6.75	0.621 (0.566,0.682)	0.844 (0.767,0.928)	0.848 (0.771,0.932)
Vertebral Fracture							
No → No	19,844	345	89,901	3.84	1 (reference)	1 (reference)	1 (reference)
No → Yes	15,652	173	71,940	2.40	0.625 (0.521, 0.750)	0.777 (0.646,0.933)	0.779 (0.648,0.936)
Yes → No	11,849	164	53,878	3.04	0.793 (0.659, 0.955)	0.892 (0.741,1.075)	0.893 (0.741,1.076)
Yes → Yes	27,429	218	123,300	1.77	0.462 (0.390, 0.547)	0.698 (0.587,0.830)	0.703 (0.591,0.836)
Hip Fracture							
No → No	19,844	48	90,631	0.53	1 (reference)	1 (reference)	1 (reference)
No → Yes	15,652	24	72,327	0.33	0.623 (0.382,1.017)	0.844 (0.515,1.381)	0.861 (0.526,1.410)
Yes → No	11,849	30	54,236	0.55	1.045 (0.662,1.649)	1.230 (0.778,1.944)	1.211 (0.766,1.916)
Yes → Yes	27,429	15	123,773	0.12	0.231 (0.129,0.412)	0.393 (0.218,0.711)	0.405 (0.224,0.732)

Model 1, crude; Model 2, adjusted for age and sex; Model 3, adjusted for age, sex, diabetes, hypertension, hyperlipidemia, chronic kidney disease, low income, and type of thyroidectomy; *Rate; incidence rates per 1000 person years

### Subgroup analyses

Subgroup analyses were performed according to patient age, sex, and surgical method (total thyroidectomy vs. lobectomy) to evaluate whether the effect of regular exercise in reducing the fracture risk differed according to clinical characteristics. The reduction in any fracture risk among those who exercised regularly before and after surgery was consistently confirmed across all subgroups except the lobectomy group (**Table 3**). The risk reduction for vertebral fractures upon the initiation of exercise was found to be significant in the high-risk groups of patients – women and total thyroidectomy patients; the adjusted hazard ratio (95% confidence interval) was 0.76 (0.62–0.93) in female group and 0.80 (0.66–0.97) in total thyroidectomy group.

**Table 3:** Subgroup Analyses.

Physical Activity Change	Subgroup analyses								
	Age			Sex			Type of thyroidectomy		
	40–64	65+	P*	Male	Female	P*	Lobectomy	Total thyroidectomy	P*
Any Fracture									
No → No	1 (reference)	1 (reference)	0.032	1 (reference)	1 (reference)	0.522	1 (reference)	1 (reference)	0.649
No → Yes	1.02 (0.91, 1.15)	0.84 (0.69, 1.04)		0.76 (0.53, 1.11)	0.99 (0.90, 1.10)		1.13 (0.88, 1.44)	0.95 (0.85, 1.06)	
Yes → No	0.93 (0.81, 1.05)	1.01 (0.83, 1.23)		0.78 (0.54, 1.15)	0.96 (0.86, 1.08)		1.03 (0.79, 1.35)	0.94 (0.83, 1.05)	
Yes → Yes	0.90 (0.81, 1.01)	0.70 (0.57, 0.86)		0.73 (0.54, 0.98)	0.87 (0.79, 0.97)		0.90 (0.72, 1.14)	0.85 (0.77, 0.95)	
Vertebral Fracture									
No → No	1 (reference)	1 (reference)	0.983	1 (reference)	1 (reference)	0.188	1 (reference)	1 (reference)	0.769
No → Yes	0.76 (0.60, 0.96)	0.82 (0.61, 1.11)		0.64 (0.36, 1.14)	0.80 (0.66, 0.97)		0.94 (0.60, 1.48)	0.76 (0.62, 0.93)	
Yes → No	0.89 (0.70, 1.13)	0.91 (0.68, 1.22)		0.44 (0.23, 0.88)	0.96 (0.79, 1.17)		1.01 (0.63, 1.62)	0.88 (0.72, 1.08)	
Yes → Yes	0.71 (0.57, 0.87)	0.72 (0.54, 0.98)		0.55 (0.35, 0.87)	0.74 (0.61, 0.89)		0.70 (0.45, 1.08)	0.72 (0.60, 0.87)	
Hip Fracture									
No → No	1 (reference)	1 (reference)	0.855	1 (reference)	1 (reference)	0.866	1 (reference)	1 (reference)	0.570
No → Yes	0.69 (0.32, 1.47)	1.03 (0.54, 1.95)		1.11 (0.30, 4.15)	0.83 (0.49, 1.42)		1.09 (0.31, 3.88)	0.83 (0.49, 1.42)	
Yes → No	1.21 (0.60, 2.41)	1.23 (0.66, 2.26)		1.22 (0.33, 4.54)	1.24 (0.76, 2.02)		2.04 (0.65, 6.35)	1.12 (0.67, 1.85)	
Yes → Yes	0.34 (0.16, 0.82)	0.46 (0.20, 1.05)		0.61 (0.18, 2.13)	0.36 (0.18,0.72)		0.19 (0.02, 1.60)	0.45 (0.24, 0.83)	

Model adjusted for age, sex, diabetes, hypertension, hyperlipidemia, chronic kidney disease, low income, types of thyroidectomy, and lifestyle factors (weight, waist circumference, smoking, drinking, and regular exercise); *P values were calculated by the interaction analysis.

## Discussion

This population-based observational study evaluated the association between physical activity and fracture risk in thyroid cancer patients who underwent thyroidectomy. We found a significant reduction in fracture risk among patients who exercised regularly both before and after surgery, compared with the sedentary group; the adjusted hazard ratio for any fracture was 0.848 (95% confidence interval 0.771–0.932). In the subgroup analysis, the risk reduction for vertebral fractures was confirmed even in patients who started their exercise after surgery: adjusted hazard ratio 0.779 (95% confidence interval 0.648–0.936). The results of this study suggest that maintaining physical activity is important for preventing fractures in thyroid cancer patients. In addition, the initiation of regular exercise could be recommended for patients with a sedentary lifestyle who undergo thyroidectomy.

Previous studies reported that TSH-suppression therapy could reduce bone mineral density (BMD), particularly in postmenopausal women (16). In a previous population-based study using claims data, a significant increase in fracture risk was not confirmed in all patients with thyroid cancer, but the patients who underwent thyroidectomy received osteoporosis treatment more often than the control group (17). In an analysis performed according to the dose of levothyroxine, a significant increase in osteoporotic fracture was confirmed in the highest quartile (more than 170mcg daily) patient group compared with the lowest quartile (< 115mcg daily) patient group (17). Vertebral fractures sometimes occur without symptoms, and the risk of fracture can be underestimated when the outcome is evaluated using only clinical fractures. Therefore, another study considered the outcome of radiologic vertebral fractures and reported a 25-fold odds ratio for fracture risk in patients with TSH suppression of less than 0.5uIU/ml, and the risk increased depending on the level and duration of TSH suppression therapy (18). In addition, high-dose levothyroxine treatment is thought to affect not only bone quantity but also bone quality in patients with thyroid cancer, which was confirmed in previous studies that examined changes in the trabecular bone score (19) and bone geometry (20). Therefore, thyroid cancer patients are considered to be a high-risk group for osteoporosis and fragility fractures, and it is important to pay attention to their bone health.

Many previous studies have evaluated the association between regular exercise and bone health. An exercise-based randomized clinical trial for postmenopausal women showed that a strength exercise program for 30 minutes twice a week could significantly increase BMD compared with the light stretching exercise for 15 minutes used in the control group (21). An exercise study of older men reported that BMD increased in proportion to the intensity of exercise (22). BMD, currently the most widely used indicator of bone health, mainly evaluates bone quantity, but fragility fractures are not related only to bone mass (23). In recent studies on skeletal health in older adults, sarcopenia, defined as a loss of muscle mass and strength, has also been suggested as a risk of fracture (24). Regular exercise could help prevent fractures by maintaining muscle mass and strength (25). In addition, functional performance decline with aging is associated with fractures (26). Improving exercise ability, such as flexibility and sense of equilibrium to cope with falls, through consistent physical activity could also help prevent fractures (27).

The amount of exercise recommended by the World Health Organization for adults is 150 minutes or more of moderate-intensity physical activity or 75 minutes or more of vigorous-intensity physical activity per week, and multicomponent exercise at least 3 times a week is recommended to prevent falls for adults aged 65 years or older (28). However, exercising with excessively high-intensity or for long times can also increase the risk of falls and associated fractures (29). A previous meta-analysis indicated that three or more exercise sessions per week can increase the risk of falls among elderly people (30). Therefore, deciding on the type and frequency of exercise needed to minimize the risk of fracture could require a personalized approach. In a previous large-scale population-based study, exercise such as daily walking or weekly running in leisure time was significantly associated with a decrease in fracture risk (31). Therefore, in our analysis of the degree of fracture risk reduction, we defined regular exercise as moderate to high intensity physical activity at least once a week. The results of this study suggest that exercise for 30 minutes once a week could be recommended for thyroid cancer patients who have undergone total thyroidectomy or are older than 65 years.

In this study, we found that not only regular physical activity before and after thyroidectomy but also regular exercise initiated after surgery was associated with a significantly reduced risk of vertebral fracture in thyroid cancer patients. However, we could not confirm an association between starting regular exercise after surgery and reducing hip fractures. Vertebral fractures are characteristically a site with little association with trauma (32), and falls due to excessive exercise increase the risk of fractures, especially in the wrist, ankle, or hip area (33, 34). Because the population included in our definition of regular exercise included patients who continued to exercise at high intensity exercise that causes falls, our failure to confirm a significant decrease in hip fracture may have been due to fractures related to strenuous exercise. In addition, hip fracture is the most severe form of osteoporotic fracture, and its incidence is low. The incidence of hip fracture in our study population was lower than that of vertebral fracture, which could also explain our inability to confirm statistical significance.

In our subgroup analyses, exercise once a week did not show a significant effect in reducing fracture risk in the lobectomy group. Compared with the total thyroidectomy group, the lobectomy group was relatively young, had fewer comorbidities (**Supplementary Table ST2**), and was at lower risk of fragility fractures. In addition, the patients who underwent lobectomy might not have taken thyroid hormone or received lower doses of levothyroxine to target mild TSH suppression (35). The low incidence of fractures in this low-risk population made it difficult to assess statistically significant changes in fracture risk. In addition, more exercise might be needed to prevent fractures in the group at low risk for falls and fractures.

The strength of this study is that it is a large-scale study using a nationwide cohort. Since the construction of the data is carried out by a national institution, the possibility of researcher bias is low. In addition, although this study was conducted as a retrospective study, efforts were made to reduce reverse causality through a longitudinal follow-up study design and a method of excluding patients previously diagnosed with fractures. Healthy people are more likely to exercise than unhealthy people, and their overall good health might protect against fractures at the same time. However, all our study subjects regularly received medical examinations and were relatively ambulatory and healthy patients. Therefore, it is considered unlikely that other disabilities, which can reduce the amount of exercise, may have affected the results. In terms of the research contents, this is a rare epidemiologic study focused on the life style in thyroid cancer survivors. Considering the generally good prognosis and long survival period, the evidence of lifestyle recommendations for thyroid cancer survivors is very important. Therefore, this study is considered to be valuable data that can provide evidence for recommendations in management of thyroid cancer survivors.

There are several limitations of this study. First, assessment of the level of physical activity using a self-declared questionnaire may be a limitation of the study, although this is the method of choice in a large population-based design. Second, the dataset used in this study consisted only of basic health examination data and did not include information related to TSH suppression or levels of bone turnover markers. For the same reason, the baseline BMD of the entire patient could not be analyzed statistically. Third, the pathological subtype and stage of thyroid cancer could not be classified due to limited data. Among thyroid cancers, medullary thyroid carcinoma does not require levothyroxine suppressive therapy. The goal of TSH suppression in differentiated thyroid cancer varies with risk of recurrence. However, the prevalence of medullary thyroid cancer in Korea is reported to be very low at 0.6% (36). In addition, lifelong supplementation with levothyroxine can frequently result in unintended suppressive therapy. Lastly, the median 4.5 years of follow up is rather a short period to evaluate fracture risk in patients in their 40s and 50s. If they were followed for a longer period, exercise might show a significant effect in the low-risk groups, such as younger patients and those treated with lobectomy.

In conclusion, the degree of physical activity and changes in physical activity were associated with fracture risk in patients treated with thyroidectomy. The results suggest that maintaining or starting regular exercise may help prevent fractures in thyroid cancer patients older than 40 years who have undergone total thyroidectomy.

## Data availability statement

The datasets presented in this study can be found in online repositories. The names of the repository/repositories and accession number(s) can be found below: https://nhiss.nhis.or.kr/bd/ay/bdaya001iv.do.

## Ethics statement

The studies involving human participants were reviewed and approved by Yeouido St.Mary’s Hospital. Written informed consent was waived because of the study protocol using anonymous data provided through public institutions.

## Author contributions

KH conceived the study. JK wrote the manuscript. KH and J-HJ. did the statistical analyses. JK, KH, J-HJ, JH, CJ, J-YH, S-WL, JL, YL, MK, H-SK, K-HS, and K-HB contributed to the interpretation of data, critically reviewed and revised the manuscript, and approved the final manuscript.
