# Supplementary material for: Physical activity and reduced risk of fracture in thyroid cancer patients after thyroidectomy — a nationwide cohort study
Source: Front Endocrinol (Lausanne). 2023 Jul 20;14:1173781. doi: 10.3389/fendo.2023.1173781 (PMC10400320; doi:10.3389/fendo.2023.1173781)
Supplement: Supplementary file 1 [file Table_1.docx]

Supplementary Table ST1. Questions related to physical activity (exercise) in the health checkup questionnaire

| Please read the questions below and circle the answer that best describes your activity in the past week.   1. In the past week, on how many days did you engage in strenuous exercise for more than 20 minutes that made you out of breath much more than usual? (For example: running, aerobics, cycling, mountain climbing, etc.)   ⓞ ① ② ③ ④ ⑤ ⑥ ⑦   1. In the past week, on how many days did you engage in moderate physical activity that made you breathe a little harder than usual for more than 30 minutes? (For example: jogging, playing doubles tennis, etc.)   ⓞ ① ② ③ ④ ⑤ ⑥ ⑦   1. In the past week, on how many days in total did you walk for at least 30 minutes, including walking for at least 10 minutes at a time? (For example: yoga, walking during commuting or leisure time, etc.)   ⓞ ① ② ③ ④ ⑤ ⑥ ⑦ |
| --- |
| In this study, individuals who marked in items ①-⑦ in question 1 or items ①-⑦ in question 2 were classified as the physically active groups (patients who exercised regularly). |

Supplementary Table ST2. Baseline characteristics of patients according to the surgical method

|  | **Total thyroidectomy** | **Lobectomy** | ***P*** |
| --- | --- | --- | --- |
| **Number** | 58,193 | 16,581 |  |
| Age (mean$\pm$sd) | 53.92±8.71 | 52.25±8.26 | <.001 |
| Sex, female (n, %) | 46,128 (79.27) | 12,660 (76.35) | <.001 |
| Height (mean$\pm$sd) | 159.71±7.82 | 160.51±7.99 | <.001 |
| Weight (mean$\pm$sd) | 62.01±10.7 | 62.28±10.78 | <.001 |
| Body-mass index (mean$\pm$sd) | 24.24±3.27 | 24.09±3.20 | <.001 |
| Waist circumference (mean$\pm$sd) | 80.00±8.96 | 79.65±8.98 | <.001 |
| Hypertension (n, %) | 20,530 (35.28) | 4,920 (29.67) | <.001 |
| Diabetes (n, %) | 6,669 (11.46) | 1,580 (9.53) | <.001 |
| Hyperlipidemia (n, %) | 16,307 (28.02) | 4,865 (29.34) | <.001 |
| Chronic kidney disease (n, %) | 2,309 (3.97) | 512 (3.09) | <.001 |
